# Supplementary material for: Environmental DNA detection and quantification of invasive red-eared sliders, Trachemy scripta elegans, in ponds and the influence of water quality
Source: PeerJ. 2019 Dec 6;7:e8155. doi: 10.7717/peerj.8155 (PMC6901006; doi:10.7717/peerj.8155)
Supplement: Supplemental Information 1 [file peerj-07-8155-s001.docx]

**Supplemental Materials**

Environmental DNA detection and quantification of invasive red-eared sliders, *Trachemy scripta elegans*, in ponds and the influence of water quality

Aozora Kakuda, Izumi Katano, Rio Souma, Mariko Nagano, Toshifumi Minamoto, and Hideyuki Doi

Table S1A Results of the water quality analysis. Not measured because of sample lost is represented by -

| Site No. | Chl. *a*（mg/L） | SS (mg/L) | PO₄-P （µmol/L) | NO₃-N （µmol/L） | TP （µmol/L） | TN （µmol/L） | DOM  (abs254nm) | TOM  (abs254nm) |
| --- | --- | --- | --- | --- | --- | --- | --- | --- |
| 1 | 0.046 | 67.20 | - | - | - | - | - | - |
| 2 | 0.008 | 9.84 | - | - | - | - | - | - |
| 3 | 0.014 | 18.52 | - | - | - | - | - | - |
| 4 | 0.058 | 47.07 | 4.777 | 42.28 | 5.541 | 31.86 | 0.101 | 0.001 |
| 5 | 0.031 | 20.00 | 0.275 | 28.03 | 0.743 | 26.69 | 0.076 | 0.026 |
| 6 | 0.006 | 11.48 | 0.344 | 22.53 | 0.608 | 23.93 | 0.056 | 0.027 |
| 7 | 0.007 | 11.64 | 0.103 | 13.03 | 3.649 | 12.55 | 0.029 | 0.007 |
| 8 | 0.052 | 50.80 | 0.481 | 56.03 | 1.689 | 35.66 | 0.139 | 0.011 |
| 9 | 0.008 | 20.67 | 0.647 | 13.66 | 0.321 | 12.71 | - | - |
| 10 | 0.044 | 37.60 | 0.344 | 37.03 | 1.419 | 30.48 | 0.087 | 0.004 |
| 11 | 0.234 | 200.40 | 1.765 | 115.85 | 3.654 | 132.00 | - | - |
| 12 | 0.026 | 22.93 | 0.206 | 22.28 | 1.216 | 13.93 | 0.053 | 0.000 |
| 13 | 0.005 | 12.90 | 1.718 | 50.53 | 2.703 | 52.90 | 0.037 | 0.002 |
| 14 | 0.022 | 22.55 | 0.309 | 28.03 | 1.014 | 19.10 | 0.069 | 0.002 |
| 15 | 0.026 | 23.40 | 0.241 | 30.53 | 0.743 | 10.83 | 0.076 | 0.000 |
| 16 | 0.006 | 14.76 | 0.241 | 36.28 | 0.946 | 18.76 | 0.093 | 0.009 |
| 17 | 0.025 | 23.25 | 0.309 | 39.03 | 0.878 | 13.93 | 0.101 | 0.000 |
| 18 | 0.054 | 122.53 | 1.203 | 79.03 | 1.149 | 24.97 | 0.205 | 0.020 |
| 19 | 0.003 | 15.64 | 3.162 | 37.78 | 3.243 | 34.28 | 0.069 | 0.011 |
| 20 | 0.007 | 15.72 | 0.893 | 35.78 | 1.486 | 25.66 | 0.066 | 0.004 |
| 21 | 0.031 | 26.30 | 0.756 | 34.78 | 1.216 | 16.69 | 0.089 | 0.004 |
| 22 | 0.014 | 34.45 | 0.447 | 22.03 | 0.473 | 9.10 | 0.055 | 0.007 |
| 23 | 0.025 | 20.05 | 0.241 | 28.78 | 0.946 | 17.72 | 0.069 | 0.003 |
| 24 | 0.007 | 100.35 | 0.447 | 39.53 | 1.419 | 21.52 | 0.100 | 0.004 |
| 25 | 0.035 | 93.80 | 0.412 | 47.78 | 1.014 | 27.03 | 0.117 | 0.017 |
| 26 | 0.006 | 21.25 | 2.096 | 50.53 | 2.703 | 30.48 | 0.126 | 0.009 |
| 27 | 0.000 | 161.85 | 0.378 | 14.03 | 1.216 | 6.34 | 0.031 | -0.001 |
| 28 | 0.040 | 48.70 | 0.275 | 28.28 | 1.554 | 16.69 | 0.059 | 0.006 |
| 29 | 0.052 | 96.05 | 0.447 | 8.78 | 1.014 | 19.45 | 0.021 | 0.010 |
| 30 | 0.058 | 23.60 | 0.034 | 16.03 | 1.149 | 6.69 | 0.042 | 0.006 |
| 31 | -0.005 | 214.85 | 1.856 | 68.78 | 3.311 | 70.14 | 0.049 | 0.002 |
| 32 | 0.048 | 48.50 | 0.756 | 21.03 | 1.959 | 23.24 | 0.036 | 0.001 |
| 33 | 0.007 | 26.80 | 0.447 | 41.53 | 1.149 | 15.31 | 0.098 | 0.016 |
| 34 | 0.046 | 28.53 | 0.275 | 42.53 | 1.014 | 18.07 | 0.102 | 0.000 |
| 35 | 0.023 | 20.95 | 0.172 | 29.53 | 0.541 | 17.03 | 0.061 | 0.001 |
| 36 | 0.020 | 21.90 | 0.123 | 49.48 | 0.576 | 34.00 | 0.058 | 0.001 |
| 37 | 0.223 | 62.13 | 0.798 | 50.48 | 1.186 | 29.56 | 0.121 | 0.005 |
| 38 | 0.013 | 11.20 | 0.061 | 31.48 | 0.373 | 10.30 | 0.074 | 0.002 |
| 39 | 0.001 | 15.25 | 0.061 | 63.48 | 0.237 | 58.81 | 0.036 | 0.013 |
| 40 | -0.001 | 16.95 | 0.245 | 94.98 | 0.203 | 91.41 | 0.027 | 0.005 |
| 41 | 0.025 | 21.35 | 0.184 | 18.48 | 0.203 | 9.19 | 0.039 | 0.007 |
| 42 | 0.124 | 30.27 | 1.471 | 44.39 | 1.410 | 28.07 | - | - |
| 43 | 0.041 | 39.73 | 1.353 | 35.85 | 0.449 | 11.29 | - | - |
| 44 | -0.002 | 16.05 | 0.798 | 34.73 | 0.373 | 19.56 | 0.055 | 0.001 |
| 45 | 0.033 | 46.25 | 0.613 | 19.73 | 0.407 | 14.74 | 0.043 | 0.013 |
| 46 | -0.002 | 26.95 | 1.104 | 70.23 | 2.068 | 108.44 | 0.049 | 0.060 |
| 47 | 0.007 | 55.10 | 1.656 | 55.73 | 1.085 | 35.85 | 0.134 | 0.025 |
| 48 | 0.381 | 61.07 | 5.399 | 77.48 | 5.153 | 41.41 | 0.185 | 0.007 |
| 49 | 0.005 | 15.45 | 0.491 | 28.23 | 0.441 | 6.96 | 0.064 | 0.001 |
| 50 | 0.008 | 127.90 | 1.902 | 32.98 | 0.305 | 28.81 | 0.031 | 0.008 |

Table S1B Continued

| Site No. | Chl. *a* （mg/L） | SS (mg/L) | PO₄-P （µmol/L) | NO₃-N （µmol/L） | TP （µmol/L） | TN （µmol/L） | DOM  (abs254nm) | TOM  (abs254nm) |
| --- | --- | --- | --- | --- | --- | --- | --- | --- |
| 51 | -0.001 | 29.10 | 1.166 | 29.48 | 0.271 | 21.78 | 0.042 | 0.006 |
| 52 | -0.001 | 40.45 | 1.104 | 34.48 | 0.305 | 28.07 | 0.047 | 0.010 |
| 53 | 0.006 | 37.80 | 0.613 | 54.48 | 0.475 | 36.59 | 0.065 | 0.003 |
| 54 | 0.008 | 27.45 | 0.798 | 61.48 | 0.644 | 28.44 | 0.115 | 0.002 |
| 55 | 0.001 | 29.70 | 0.429 | 35.23 | 0.407 | 18.81 | 0.083 | 0.014 |
| 56 | 0.252 | 51.93 | 0.613 | 26.48 | 0.508 | 16.22 | 0.057 | 0.002 |
| 57 | 0.045 | 18.93 | 0.245 | 40.98 | 0.441 | 15.11 | 0.097 | 0.001 |
| 58 | 0.040 | 21.15 | 0.368 | 25.73 | 0.576 | 11.41 | 0.060 | 0.008 |
| 59 | 0.162 | 105.00 | 0.307 | 80.48 | 0.915 | 36.22 | 0.197 | 0.016 |
| 60 | 0.045 | 37.67 | 0.429 | 43.98 | 1.322 | 22.15 | 0.103 | 0.005 |
| 61 | 0.088 | 40.33 | 0.307 | 43.48 | 1.186 | 25.48 | 0.103 | -0.001 |
| 62 | 0.044 | 23.90 | 0.061 | 35.48 | 0.746 | 16.22 | 0.076 | 0.000 |
| 63 | 0.057 | 31.10 | 0.245 | 47.73 | 1.153 | 19.19 | 0.110 | 0.000 |
| 64 | 0.105 | 32.95 | 0.613 | 56.73 | 0.508 | 33.63 | 0.142 | 0.019 |
| 65 | 0.882 | 381.71 | 0.429 | 56.23 | 0.746 | 26.96 | 0.135 | 0.002 |
| 66 | 0.014 | 13.10 | 0.368 | 27.48 | 0.339 | 6.59 | 0.066 | 0.000 |
| 67 | 0.043 | 23.50 | 0.184 | 36.23 | 0.441 | 10.67 | 0.084 | 0.001 |
| 68 | 0.138 | 85.10 | 0.060 | 50.38 | 0.780 | 24.10 | 0.121 | -0.001 |
| 69 | 0.089 | 42.15 | 0.357 | 49.87 | 1.064 | 20.31 | 0.104 | 0.001 |
| 70 | 0.064 | 26.05 | 0.298 | 30.13 | 0.780 | 23.07 | 0.065 | 0.009 |
| 71 | 0.017 | 19.53 | 1.412 | 25.37 | 0.897 | 15.93 | - | - |
| 72 | 0.034 | 28.00 | 0.417 | 30.13 | 0.638 | 20.66 | 0.051 | 0.002 |
| 73 | 0.011 | 13.70 | 0.536 | 36.03 | 0.071 | 44.10 | 0.016 | -0.002 |
| 74 | 0.007 | 14.60 | 0.417 | 29.10 | 0.709 | 15.48 | 0.050 | -0.001 |
| 75 | 0.006 | 21.80 | 0.119 | 22.95 | 0.284 | 8.24 | 0.050 | -0.001 |
| 76 | 0.006 | 29.76 | 0.298 | 48.85 | 0.213 | 47.55 | 0.015 | 0.005 |
| 77 | 0.007 | 21.64 | 0.655 | 54.23 | 0.142 | 49.62 | 0.047 | 0.003 |
| 78 | 0.011 | 15.32 | 0.952 | 33.72 | 0.213 | 15.48 | 0.081 | 0.006 |
| 79 | 0.004 | 13.28 | 0.417 | 48.85 | 0.567 | 41.00 | 0.056 | 0.003 |
| 80 | 0.001 | 14.64 | 0.595 | 23.72 | 0.142 | 16.17 | 0.040 | -0.002 |
| 81 | 0.001 | 20.04 | 0.476 | 45.26 | 0.071 | 30.31 | 0.050 | -0.005 |
| 82 | 0.004 | 25.28 | 0.238 | 62.69 | 0.142 | 46.52 | 0.062 | -0.002 |
| 83 | 0.025 | 25.60 | 0.655 | 35.51 | 0.496 | 27.55 | 0.077 | -0.002 |
| 84 | 0.004 | 45.40 | 2.059 | 74.15 | 0.513 | 47.00 | - | - |
| 85 | 0.014 | 44.85 | 2.321 | 43.72 | 2.128 | 185.48 | 0.106 | 0.065 |
| 86 | 0.018 | 65.75 | 2.824 | 41.95 | 1.218 | 201.64 | - | - |
| 87 | 0.016 | 17.48 | 0.893 | 37.82 | 0.213 | 16.52 | 0.090 | 0.003 |
| 88 | 0.006 | 21.12 | 0.774 | 25.51 | 0.355 | 12.72 | 0.057 | 0.001 |
| 89 | 0.012 | 87.60 | 0.893 | 21.92 | 0.426 | 25.83 | 0.033 | 0.001 |
| 90 | 0.002 | 18.04 | 0.595 | 19.36 | 2.128 | 16.52 | 0.032 | -0.002 |
| 91 | 0.006 | 9.88 | 0.765 | 20.24 | 0.385 | 14.14 | - | - |
| 92 | 0.003 | 14.28 | 0.714 | 24.23 | 0.496 | 19.28 | 0.036 | 0.004 |
| 93 | 0.008 | 20.20 | 0.952 | 31.41 | 1.915 | 23.76 | 0.068 | 0.004 |
| 94 | 0.002 | 12.28 | 0.298 | 22.18 | 0.567 | 14.10 | 0.050 | 0.000 |
| 95 | 0.011 | 30.08 | 0.595 | 19.10 | 0.284 | 12.72 | 0.040 | -0.003 |
| 96 | 0.031 | 17.40 | 0.595 | 32.44 | 0.709 | 21.34 | 0.075 | 0.006 |
| 97 | 0.004 | 21.48 | 1.131 | 15.51 | 0.142 | 9.97 | 0.036 | 0.000 |
| 98 | 0.034 | 41.13 | 0.893 | 32.95 | 0.709 | 29.28 | 0.069 | 0.000 |
| 99 | 0.019 | 21.80 | 0.357 | 30.38 | 0.638 | 15.14 | 0.070 | -0.002 |
| 100 | 0.015 | 26.88 | 0.595 | 31.15 | 0.284 | 15.14 | 0.071 | 0.007 |

Table S2 Linear regression slopes with a ± 95% confidence interval, SE, t values, p values, and VIFs for the relationships between the water quality analysis factors and eDNA concentration in the ponds. Turtles represent estimated number of red-eared sliders.

| Water　quality analysis | slope | SE | *t* value | *p* value | VIF |
| --- | --- | --- | --- | --- | --- |
| Chl. *a* | -19.410 | 6.073 | -3.196 | 0.019 | 4.391 |
| SS | 0.016 | 0.019 | 0.831 | 0.438 | 6.329 |
| PO₄-P | -0.483 | 0.503 | -0.960 | 0.374 | 8.871 |
| NO₃-N | 0.009 | 0.037 | 0.247 | 0.813 | 49.731 |
| TP | 0.037 | 0.112 | 0.325 | 0.756 | 1.870 |
| TN | -0.0007 | 0.027 | -0.028 | 0.978 | 78.288 |
| DOM | 0.208 | 10.070 | 0.019 | 0.985 | 13.261 |
| TOM | -7.539 | 18.660 | -0.404 | 0.700 | 16.621 |
| Turtles | 0.0004 | 0.00005 | 7.577 | 0.000 | 1.801 |
| Intercept | 8.981 | 0.4172 | 21.528 | 0.000 | - |


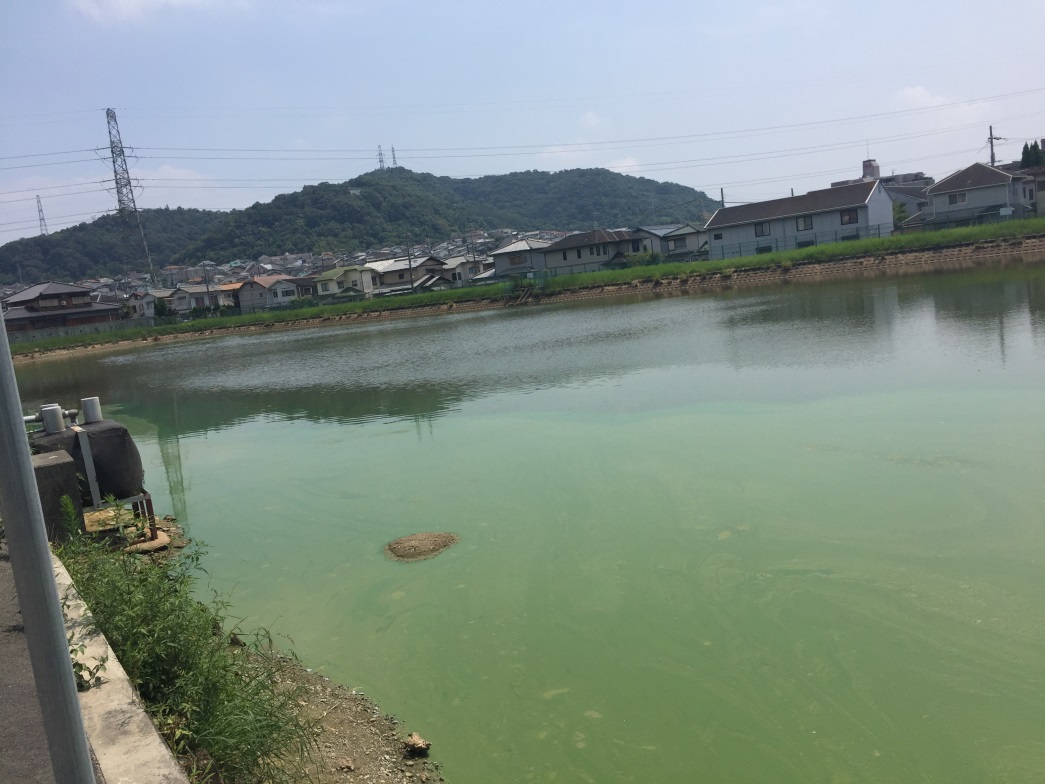


Figure S1 Pond No. 11


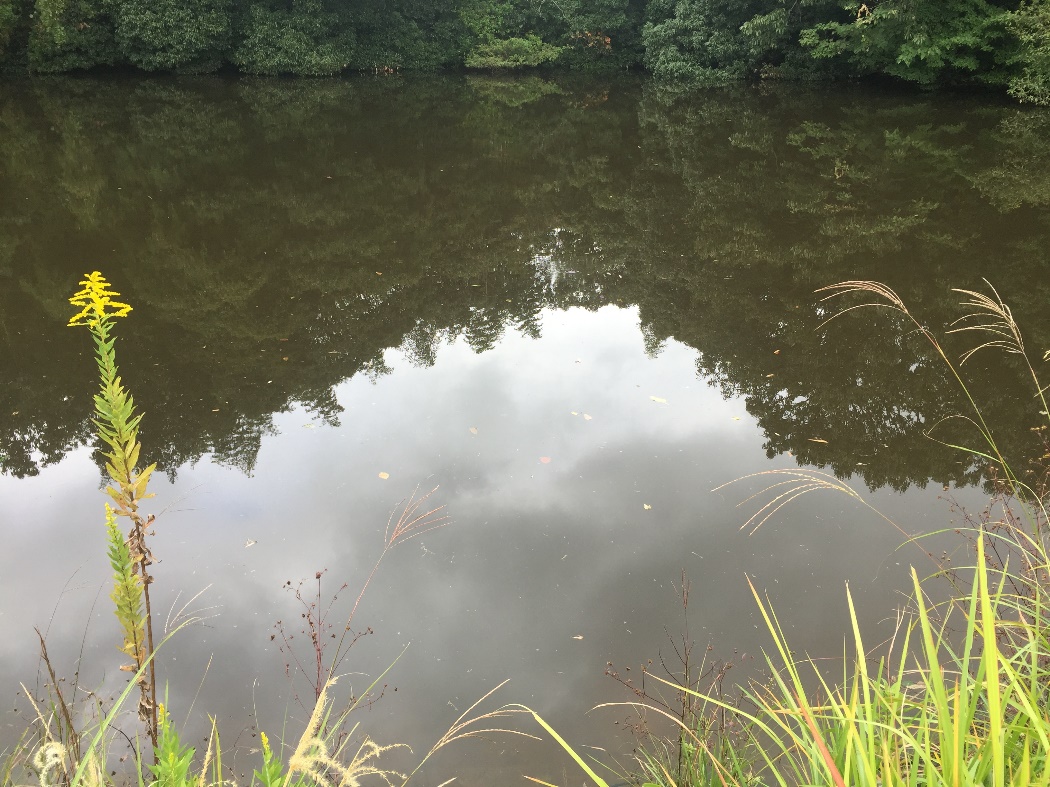


Figure S2 Pond No. 33


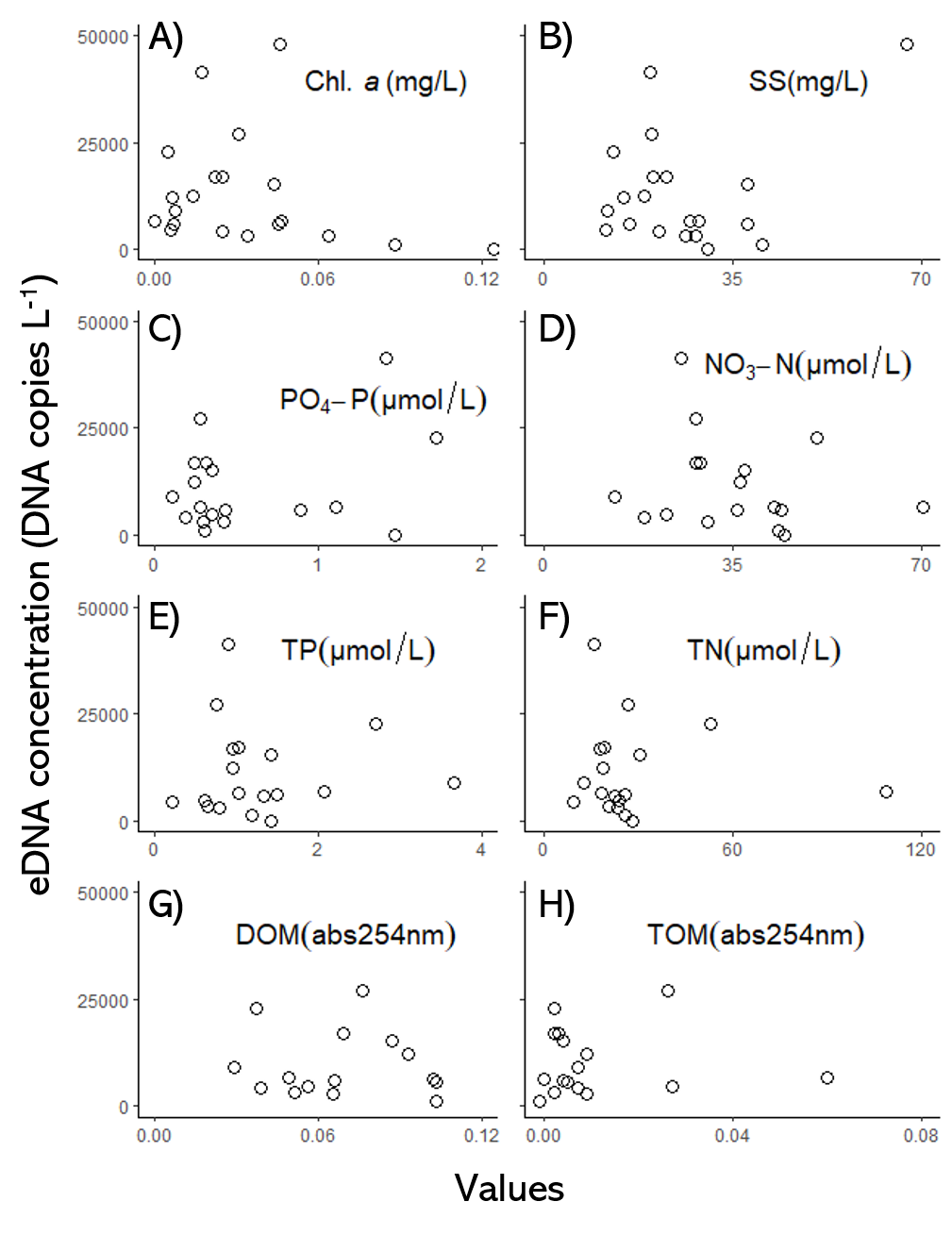


Figure S3 Relationship between each water quality factor and the eDNA concentration of the red-eared slide in the ponds, which is not log-transformed (A: Chl. *a*, B: SS, C: PO_4_-P, D: NO_3_-N, E: TP, F: TN, G: DOM, H: TOM).
